# Noise or signal? Spontaneous activity of dorsal horn neurons: patterns and function in health and disease

**DOI:** 10.1007/s00424-024-02971-8

**Published:** 2024-06-01

**Authors:** Javier Lucas-Romero, Ivan Rivera-Arconada, Jose Antonio Lopez-Garcia

**Affiliations:** 1 Department of Systems Biology, University of Alcala, 28805 Madrid, Spain; 2 Department of Physical Therapy, Washington University School of Medicine, Washington University in St. Louis, St. Louis, MO 63108 USA; 3 Departamento de Biologia de Sistemas, Edificio de Medicina, Universidad de Alcala, Ctra. Madrid-Barcelona, Km 33,600, 28805 Alcala de Henares, Madrid Spain

**Keywords:** Spike trains, Firing pattern, Spinal cord, Sensory processing

## Abstract

Spontaneous activity refers to the firing of action potentials by neurons in the absence of external stimulation. Initially considered an artifact or “noise” in the nervous system, it is now recognized as a potential feature of neural function. Spontaneous activity has been observed in various brain areas, in experimental preparations from different animal species, and in live animals and humans using non-invasive imaging techniques. In this review, we specifically focus on the spontaneous activity of dorsal horn neurons of the spinal cord. We use a historical perspective to set the basis for a novel classification of the different patterns of spontaneous activity exhibited by dorsal horn neurons. Then we examine the origins of this activity and propose a model circuit to explain how the activity is generated and transmitted to the dorsal horn. Finally, we discuss possible roles of this activity during development and during signal processing under physiological conditions and pain states. By analyzing recent studies on the spontaneous activity of dorsal horn neurons, we aim to shed light on its significance in sensory processing. Understanding the different patterns of activity, the origins of this activity, and the potential roles it may play, will contribute to our knowledge of sensory mechanisms, including pain, to facilitate the modeling of spinal circuits and hopefully to explore novel strategies for pain treatment.

## Introduccion

The central nervous system is a complex network of neural circuits responsible for receiving, processing, and transmitting sensory information. The dorsal horn of the spinal cord is an intricate region that acts as a relay and integration point for proprioceptive, tactile, thermal, visceral, and nociceptive information. Virtually all types of primary afferent neurons form synaptic connections in this region [1, 20, 24], constituting the major source of input to the dorsal horn. Neurons in this area can be broadly classified as local interneurons [90], whose synaptic targets are circumscribed to other neurons in the spinal cord, including the ventral horn, or as projection neurons, which have long axons reaching various intra- and supraspinal areas located in the medulla, the mesencephalon, and thalamus [95]. These in turn relay information to many brain areas such as the somatosensory, cingulate, and insular cortices as well as subcortical areas, which include the hypothalamus and the amygdala [94]. In addition, the dorsal horn receives abundant intraspinal and supraspinal innervation that modulates sensory information processing [63]. Intraspinal innervation comes from different segments of the cord and descending pathways originate at various points in the brainstem, the somatosensory cortex, and other brain areas.

Neurons in the dorsal horn of the spinal cord constitute a heterogeneous network in which several authors have attempted to distinguish neuronal populations according to different parameters such as functional phenotype, molecular identity, morphology, RNA expression, or electrophysiological profile [2, 34, 68, 80]. In this context, spontaneous activity is yet another valuable feature that could reflect relevant properties of neurons in this area and should not be overlooked.

Spontaneous activity, also known as baseline, resting, or ongoing activity, refers to the generation of action potentials in the absence of an identifiable external stimulus. Classical studies from the literature treated this activity as if it was indeed noise without any significant physiological relevance and consequently subtracted from responses to peripheral stimulation [84]. Although direct reference to ongoing activity as neuronal noise is scarce, in many reports of spinal cord neurons, background activity is not mentioned or mentioned but not analyzed or quantified. This is an additional indication that spontaneous activity has been historically ignored, undervalued, or treated as devoid of physiological relevance.

The studies discussed below raise the possibility that this activity has a relevant physiological role from development to actual information processing during adulthood. This is in line with recent investigations using functional magnetic resonance imaging of the brain in which the “resting state” receives a growing interest to explain brain function and disease [60].

In this review article, we examined the spontaneous activity of dorsal horn neurons from different perspectives. A historical review of data unveils a variety of activity patterns described by authors using different technical approaches and animal species. We present a rational classification of all this variance to unify criteria and aid future work.

The mechanisms involved in the generation of such activity are also discussed in depth with a focus on intrinsic membrane properties of subsets of neurons as described in more recent articles. Here we also propose a model to explain how activity from a few spontaneously active neurons may spread through the dorsal horn.

Finally, potential functional and clinical implications of the spontaneous activity of dorsal horn neurons are considered on the basis of the few publications that address this issue and a certain dose of speculation. We hope that this review may help to better the understanding of spontaneous activity and to encourage and facilitate future work in this field that keeps some intriguing and so far, insufficiently explored questions.

## Methods

A literature search was performed using the PubMed database looking for relevant keywords or phrases in titles and abstract sections. A first general search was made using the search terms “spinal cord AND/OR dorsal horn neurons AND spontaneous activity.” Titles and abstracts of articles found were screened for relevant results. Articles that included quantitative data on spontaneous activity or a description of different patterns of activity or a discussion on generation mechanisms or physiological function were selected for full analysis. An additional general search using the query “ionic channels OR ionic currents AND spontaneous activity OR spontaneous firing” was performed to discuss similarities and particularities of membrane mechanisms used to produce spontaneous activity in different areas of the CNS. This first search allowed us to build a precise structure for the present revision in different sections. Then, a second more refined search was performed using search terms corresponding to the main sections of the manuscript. A third refinement of the search was performed searching for names of authors that appeared frequently in previous searches to ensure that the work performed by relevant groups was considered in full. Additional references were found by analyzing the literature selected in previous searches and following the links to cited work.

## Results

### Patterns of spontaneous activity reported in dorsal horn neurons

Descriptions of spontaneous firing from neurons of the dorsal horn have been made using a variety of animals and experimental preparations, all of them factors that may introduce variation in results and therefore deserve mention and careful treatment throughout the text. Classical preparations were made in vivo with decerebrated, spinalized, or anesthetized cats or rats. Decerebrated preparations remove the brain after a midcollicular transection under anesthesia. This leaves the spinal cord without some descending control and allows eliminating the anesthesia during the experiments. Spinalization is a procedure that involves spinal cord transection at any given level, leaving the caudal segments without descending control but experiments are performed under anesthesia since the rostral structures are not removed. Some authors performed spinalization after decerebration (see for example [89]). The third commonly used procedure involved in vivo preparations of animals with intact nervous system under anesthesia. All these preparations required extensive and intensive surgery and delicate maintenance procedures and allow for extracellular recordings although some intracellular observations have been reported.

More recently, ex vivo preparations have become popular. In these, the spinal cord is extracted under anesthesia from young rats or mice (in most cases under one month of age) and kept in artificial cerebrospinal fluid at room temperature. These preparations keeping the entire cord or slices of it do not require the use of anesthesia during the experiments and have all its natural inputs removed (afferent and descending). In a few cases, more sophisticated in vitro preparations have been designed to include the brainstem [25] or the periphery [32, 51] attached to the cord. In most occasions, in vitro preparations have been used in combination with intracellular electrodes introducing yet another factor of variation which is the composition of the electrode internal fluid.

Despite these technical differences, the results reported tend to show the existence of some persisting coincidences that define patterns of cellular behavior. However, for the remainder of this section, the preparations used in the different experiments will be explicitly mentioned.

The first reports of spontaneous activity in individual dorsal horn neurons were made in the 1950s [29, 31, 37]. Early experiments using mostly single unit extracellular electrodes coupled to in vivo preparations of decerebrated or spinalized cats, showed that as many as 75% of recorded neurons had spontaneous activity, which came in different patterns. Some neurons fired action potentials at irregular intervals, whereas others fired rhythmically. Additionally, some neurons were described as firing bursts of action potentials [37]. Most of them responded to mechanical stimulation of their receptive fields in the skin with an increase or a decrease of their basal activity.

In the 1970s, recordings from lumbar dorsal horn neurons of spinalized cats showed that low threshold and wide dynamic range neurons exhibited spontaneous activity, although in different proportions [30]. This was taken as an indication that spontaneous activity exists in spinal circuits subserving different sensory modalities. Brown et al. showed that neurons with spontaneous activity have greater receptive fields in the skin than silent neurons in decerebrated and spinalized cats [15]. Tapper et al., using the same preparation, described two different patterns of spontaneous activity with inter-spike interval distributions following Poisson and bimodal types [89] (the first corresponding to irregular neurons and the second to neurons firing bursts of action potentials). Further, the authors proposed that spontaneous activity of dorsal horn neurons arises within the spinal cord since it persists after spinalization and denervation of the cord.

**Fig. 1:**
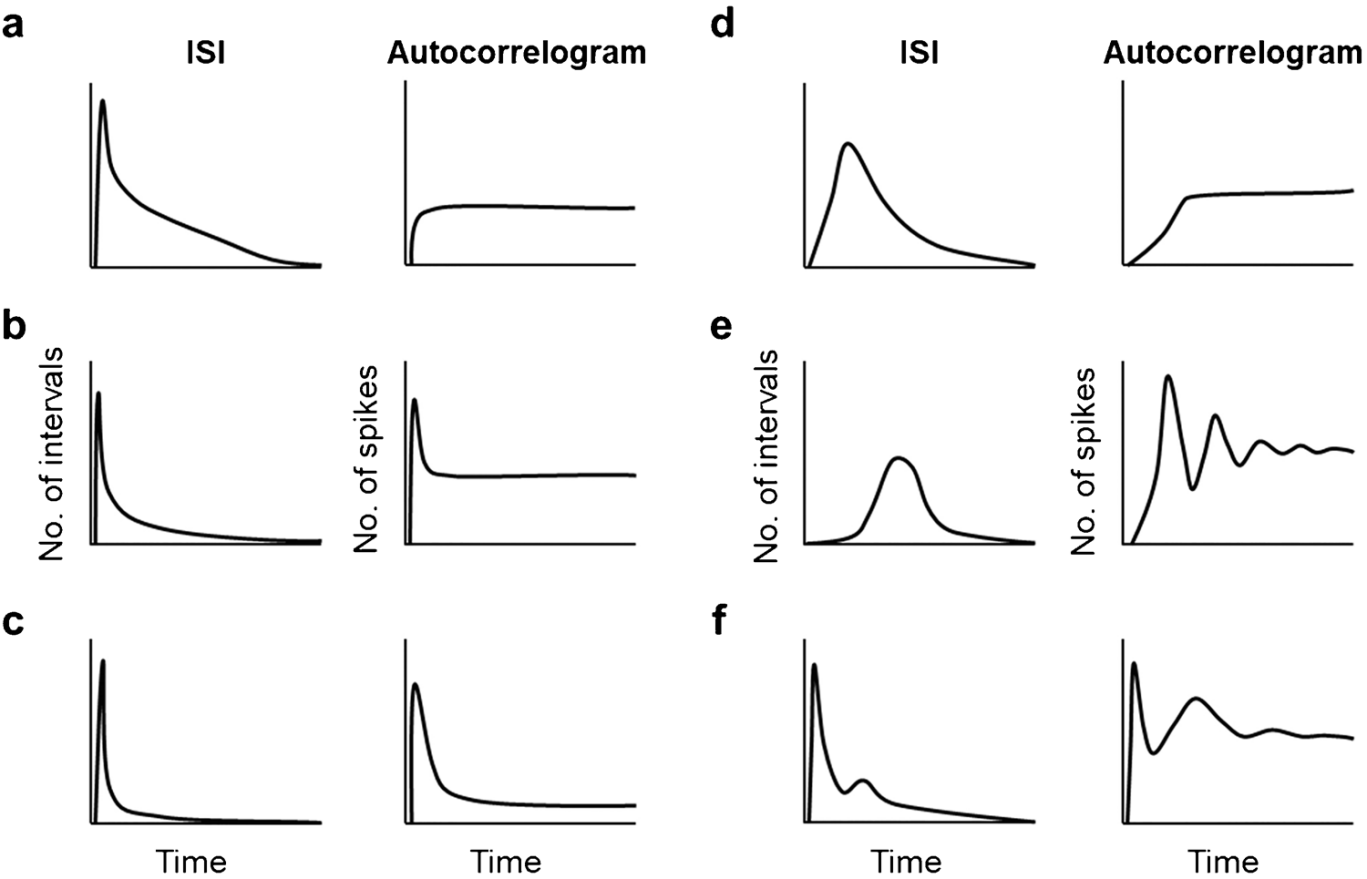
Previous classification of spontaneous firing patterns. Idealized inter-spike intervals (ISI) and auto-correlograms corresponding to the different patterns of spontaneous activity proposed by Brown et al. (1979) [14]. Graphs from real data can be found in the original publication. **a** Poisson-like distribution with a flat auto-correlogram. **b** and **c** Poisson-like correlograms with initial peaks. **d** and **e** Gaussian distributions and different degrees of regularity. **f** Bimodal frequency distribution and auto-correlogram of a neuron firing bursts at regular intervals

Brown et al. classified spontaneous activity of dorsal horn neurons in different patterns according to inter-spike intervals (ISI) and auto-correlograms [14] (see Fig. 1). Data were obtained from lumbar neurons recorded in spinalized cats, mostly located in laminae IV–VI. They described six different types of distributions of action potentials along the time axis. The first three had a Poisson-like distribution of inter-spike intervals and flat auto-correlograms, indicating irregular activity, but they could show an initial peak of variable amplitude, indicating a tendency to fire action potentials grouped in bursts. Two more ISI distributions were of the Gaussian type, with a dominating frequency and different degrees of basal activity. These two types correspond to neurons firing at regular or irregular intervals. Finally, they described a bimodal distribution corresponding to neurons firing bursts of spontaneous activity at regular intervals.

Cervero et al. studied the spontaneous activity of *substantia gelatinosa* neurons using spinalized cats as well [17]. As many as 85% of these neurons had a high frequency (5–10 Hz) irregular spontaneous activity and inhibitory responses to peripheral stimulation, that is, they stopped firing in response to light touch (inverted class 1) or to high intensity (inverted class 2) stimulation. A third type of neuron showed slightly excitatory responses to light touch and inhibitory responses to painful stimuli (inverted class 3). This pattern of spontaneous activity together with “inverted” evoked activity was not found in deeper dorsal horn neurons, suggesting a heterogeneous distribution of patterns. The authors proposed that these neurons could be inhibitory and their spontaneous activity would maintain deeper projection neurons silent in the absence of stimulation, allowing their activation upon arrival of stimuli from the periphery. Inverted neurons were reported latter on using in vitro preparations of the rat and mouse spinal cords [26, 51, 57, 79]; however, most of these neurons reported in vitro showed regular spontaneous activity. A recent work performed in the in vitro brainstem-cervical cord preparation coupled to whole cell recordings of Lamina I neurons reports a proportion of inhibitory neurons identified by pax2 that have rhythmic firing modulated by synaptic inputs [25]. Zhang et al. also reported a population of GAD67^+^ GABA-ergic neurons with regular firing of single spikes in in vitro mice cords [101]. Spontaneous activity together with inhibitory responses to peripheral stimulation had been reported in anesthetized rhesus monkeys as well [93], suggesting a general mechanism with a function rather than an experimental artifact.

Most neurons recorded in the previous studies were not identified. Several studies from the Willis laboratory focused on identified spinothalamic projecting neurons of anesthetized monkeys located in lumbar LI–VI [27, 88, 98]. They first reported irregular, regular unimodal, and regular bimodal patterns and latter on proposed a more systematic classification. In the latter, spontaneous neurons were labeled as SP1 (neurons with a moderately regular spontaneous activity devoid of short interval bursts of spikes), SP2 (neurons with high-frequency bursts of spikes), and SP3 (neurons with combined properties of the other types showing high frequency bursts at moderately regular intervals). They attributed the patterns observed to a mixture of convergent afferent input and intrinsic membrane properties of the neurons concluding that “intrinsic process may have profound implications for the coding function of STT neurons,” with SP1 neurons better suited to code slowly changing stimuli (nociceptive) and SP2 and 3 to transmit information from rapidly changing stimuli (tactile). An important contribution of these studies is the finding of spontaneous activity in projection neurons.

Sandkühler and Eblen-Zajjur studied spontaneous activity in lumbar dorsal horn neurons of anesthetized rats [84]. They used the term “rhythmicity” for events that occurred at regular intervals and distinguished between rhythmic (RN) and non-rhythmic neurons (NRN), finding that RN were mostly interneurons, whereas neurons projecting beyond cervical segments were NRN. Further, authors reserved the term burst-like for neurons with brief trains of high frequency spiking. With these two categories they showed auto-correlograms and inter-spike interval distributions corresponding to four patterns of spontaneous activity that included rhythmic bursting neurons, rhythmic neurons with single spikes, irregular bursting neurons and irregular neurons with single spikes.

Seagrove et al. and Rojas-Piloni et al. recorded spontaneous activity of LI-II neurons from anaesthetized rats using extracellular recordings [78, 86]. They reported a particular pattern of activity characterized by the firing of spikes in duplets or triplets with very brief inter-spike intervals (~ 2 ms). Duplets came at irregular intervals. These neurons appeared to receive nociceptive input and to increase their firing frequency after intraplantar administration of carrageenan while maintaining firing in duplets.

The Baccei group obtained intracellular recordings from LI-II interneurons as well as from spino-parabraquial and spino-periaqueductal neurons using in vitro preparations of the rat spinal cord containing lumbar segments [44, 47]. They described three types of neurons, including irregular, tonic, and bursting neurons, and focused their study on the later “oscillatory burst firing” also referred to as pacemakers because bursts came at regular intervals. These neurons were characterized as a class of glutamatergic interneurons with the intrinsic capacity to produce rhythmic activity. Later on, several works from the Safronov group performed on in vitro preparations of rats reported the tonic type pattern in Lamina I in local interneurons but not in projection neurons at different levels of the spinal cord and in the medulla [25, 26, 56–58]. These latter authors did not report bursting neurons in Lamina I.

Our laboratory obtained some preliminary data using multi-electrode arrays (MEAs) coupled to a longitudinal slice of the dorsal lumbar area of the cord of mice [79] in which we could observe different patterns of spontaneous activity as previously reported in in vivo preparations of different animal species. Both regular and irregular patterns were found as well as single spike and bursting neurons. More recent data from our group using larger MEAs and recording from larger samples of neurons demonstrated the existence of all possible combinations of these two variables (regular vs irregular and bursting vs single spike), and in addition, different types of bursts were detected, including fast, slow, and mixed bursts [54]. Fast bursts included typically 2–4 spikes fired at high intra-burst frequencies (> 100 Hz). Slow bursts included a large and variable number of spikes (typically 20–50) fired at lower intra-burst frequencies (< 20 Hz). Mixed bursts included large and variable number of spikes fired at high frequency at the beginning and then adapting to lower frequencies with time.

An additional contribution of our studies using MEAs is the observation that spontaneous activity in sets of dorsal horn neurons does not seem to be randomly distributed along the time axis since neurons tend to coordinate their behavior to produce “population bursts.” Population bursts are brief events (~ 200 ms) of simultaneous activity occurring at irregular intervals and concentrating action potentials from neurons exhibiting different patterns of activity [53]. The existence of these evens suggests that dorsal horn neurons are connected into circuits that coordinate their activity. Neurons with irregular patterns of spontaneous activity are major contributors toward these population events.

### Proposed classification of spontaneous activity patterns

**Fig. 2:**
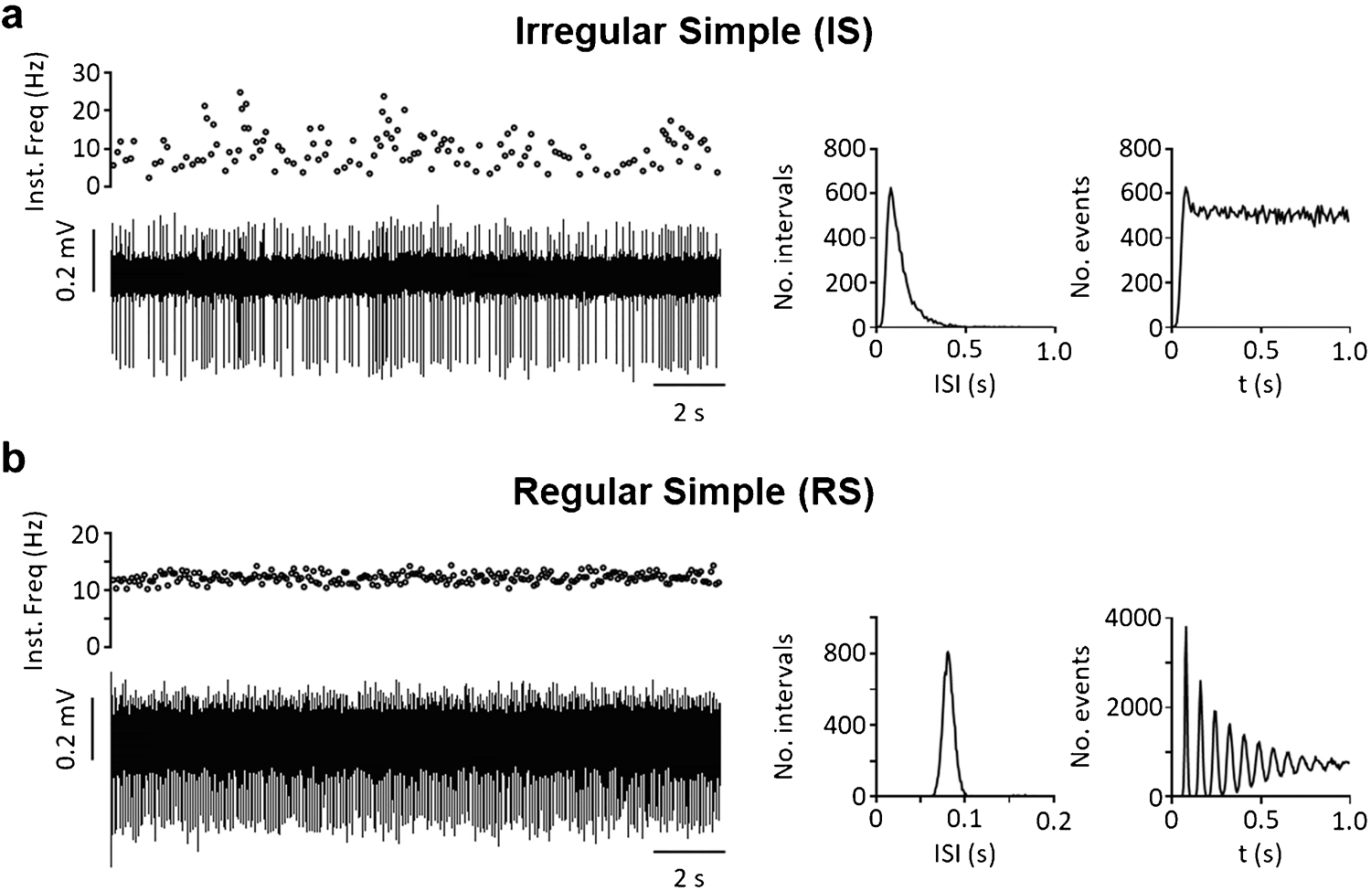
Single spike patterns. **a** shows an irregular pattern with single spikes. The original recording from a MEA is shown in the left lower panel. The corresponding instantaneous frequency (Inst. Freq) is shown above. Graphs to the right and far right show ISI distribution and auto-correlogram for this neuron. **b** shows a regular pattern with single spikes. Panels arranged as in (a)

**Fig. 3:**
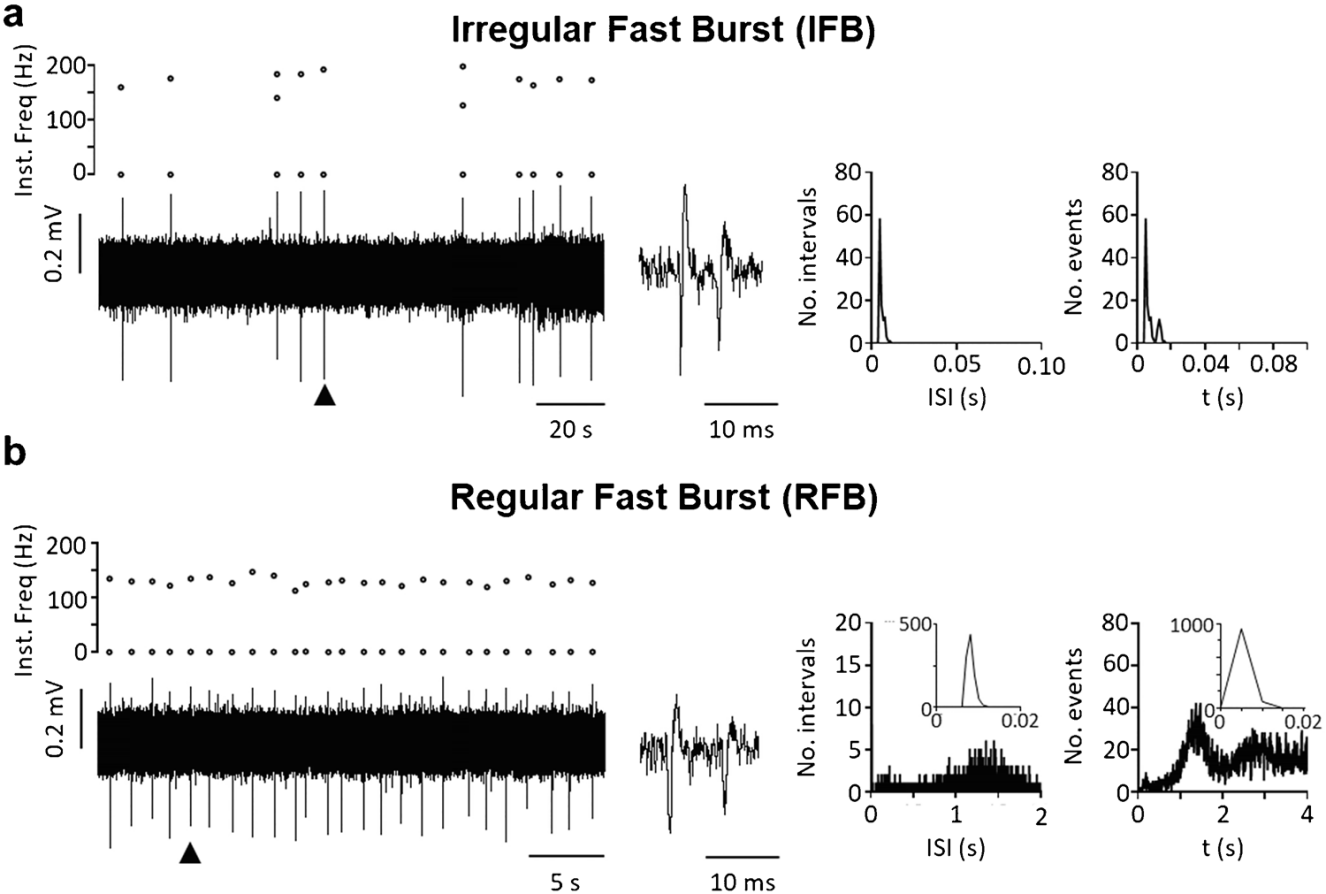
Fast burst patterns. Irregular (**a**) and regular (**b**) fast burst patterns from two dorsal horn neurons. Original extracellular recordings and graphs arranged as in Fig. 2. Middle panels show duplets fired by each neuron. Insets in ISI and auto-correlogram graphs in (b) show early peaks generated by the second spike of the duplet

**Fig. 4:**
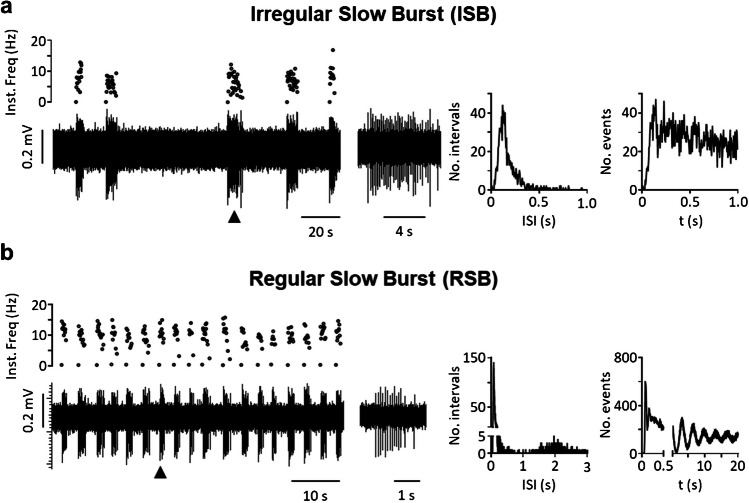
Slow burst patterns. Irregular (**a**) and regular (**b**) slow burst patterns from two different dorsal horn neurons. Original extracellular recordings and graphs arranged as in previous figures. Middle panels show bursts produced by each neuron

**Fig. 5:**
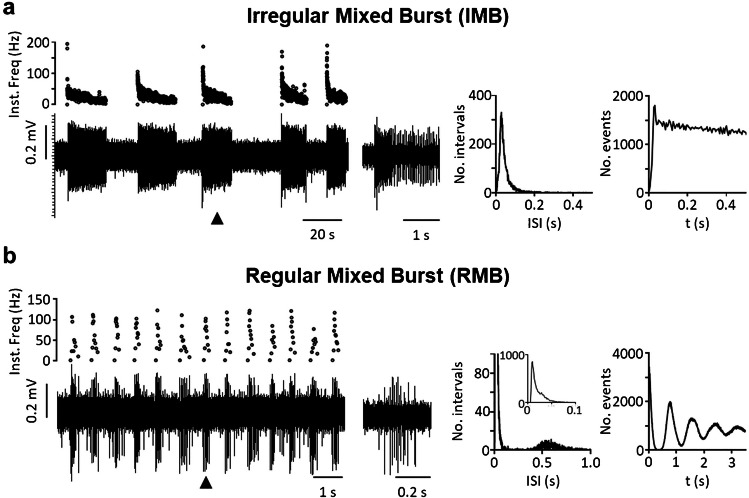
Mixed burst patterns. Irregular (**a**) and regular (**b**) mixed burst patterns from two different dorsal horn neurons. Original extracellular recordings and graphs arranged as in previous figures. Middle panels show bursts produced by each neuron. Note the initial high firing frequency and the progressive decay

Taking into account the variables most frequently mentioned by authors (regular vs irregular firing; single spike vs burst firing) as well as a new variable (intra-burst firing frequency), we were able to characterize eight different patterns of spontaneous activity that we propose as a standard to help classify neurons from past and future studies. The classification includes four types of irregular neurons—i.e., irregular single spike (IS), irregular fast burst (IFB), irregular slow burst (ISB), irregular mixed burst (IMB)—as well as their regular counterparts (RS, RFB, RSB, and RMB). Table 1 shows the main traits of each type, the frequency with which each type has been found in our in vitro experiments, and references to previous reports of similar forms of activity as discussed above. Figures 2, 3, 4 and 5 show original recordings from the different neurons obtained from our laboratory.

**Table 1:** Firing patterns classification

Firing patterns	Single	Fast burst	Slow burst	Mixed burst
Irregular neurons	IS	IFB	ISB	IMB
No. Neurons	306	130	34	14
% total	52.58%	22.34%	5.84%	2.40%
Mean firing freq. (Hz)	0.96 ± 0.09	0.41 ± 0.08	1.49 ± 0.23	2.62 ± 0.47
Mean intra-burst freq. (Hz)	–	119.9 ± 3.2	15.4 ± 2.3	37.3 ± 3.8
Mean inter-burst freq. (Hz)	–	1.70 ± 0.26	0.27 ± 0.06	0.36 ± 0.12
Mean spikes/burst	–	2.71 ± 0.11	30.84 ± 8.24	34.07 ± 13.78
Mean burst duration (s)	–	0.022 ± 0.001	7.30 ± 1.86	2.60 ± 0.68
CV for classification	1.27 ± 0.03	1.02 ± 0.04	0.81 ± 0.08	0.69 ± 0.06
CV intra-burst	–	0.63 ± 0.02	0.95 ± 0.05	1.32 ± 0.17
Previously reported	[7, 13, 14, 17, 29, 31, 37, 44, 53, 54, 58, 79, 84, 88, 93, 98]	[7, 14, 37, 59, 84, 87–89, 98]		[53, 54, 79]
		[31, 53, 54, 78, 79, 86]	[53, 54, 79]	
Regular neurons	RS	RFB	RSB	RMB
No. Neurons	64	5	27	2
% total	10.10%	0.86%	4.64%	0.34%
Mean firing freq. (Hz)	5.76 ± 0.35	7.73 ± 5.04	3.74 ± 0.49	12.56 ± 0.33
Mean intra-burst freq. (Hz)	–	115.0 ± 16.2	15.9 ± 1.8	51.90 ± 11.22
Mean inter-burst freq. (Hz)	–	5.21 ± 4.21	0.47 ± 0.10	0.74 ± 0.55
Mean spikes/burst	–	3.28 ± 0.94	43.97 ± 17.48	43.47 ± 33.13
Mean burst duration (s)	–	0.18 ± 0.15	5.73 ± 1.83	1.51 ± 1.30
CV for classification	0.31 ± 0.01	0.40 ± 0.04	0.34 ± 0.02	0.22 ± 0.06
CV intra-burst	–	1.11 ± 0.42	1.01 ± 0.08	0.65 ± 0.11
Previously reported	[7, 14, 29, 37, 44, 53, 54, 58, 79, 84, 98, 101]	[7, 14, 29, 84, 88, 89, 98]		[53]
		[53, 54]	[44, 53, 54]	

Firing patterns recorded in spontaneously active neurons in the dorsal horn of the spinal cord. Data was pooled from previous publications [53, 54] and analyzed with an in house-developed software available online at: https://github.com/Lucas-Romero-J/App_Spike_Train_Analysis_V3

^*^IS Irregular single spike, *IFB irregular fast burst, *ISB irregular slow burst, *IMB irregular mixed burst, *RS regular single spike, *RFB regular fast burst, *RSB regular slow burst, *RMB regular mixed burst, *CV coefficient of variation

In our experience, irregular patterns are about fourfold more frequently recorded than regular ones. Mixed bursting neurons (regular and irregular) are rare and have not been reported by other laboratories. Whether these patterns are real or artifactual remains to be elucidated. Additional patterns may be possible considering, for example, detailed analysis of factors such as intra-burst frequency [54]. However, we believe that this classification is sufficiently comprehensive and that types listed are likely to be representative of the physiological variability given the wide range of experimental conditions and animal species in which they have been reported.

A precise regional distribution of neurons with different patterns has not been established. While some particular types could be overrepresented in a specific area (i.e., inverted neurons) [17], there is no substantial evidence indicating clear segregation of patterns by location, although regular firing neurons may be more common in deeper layers [53].

**Fig. 6:**
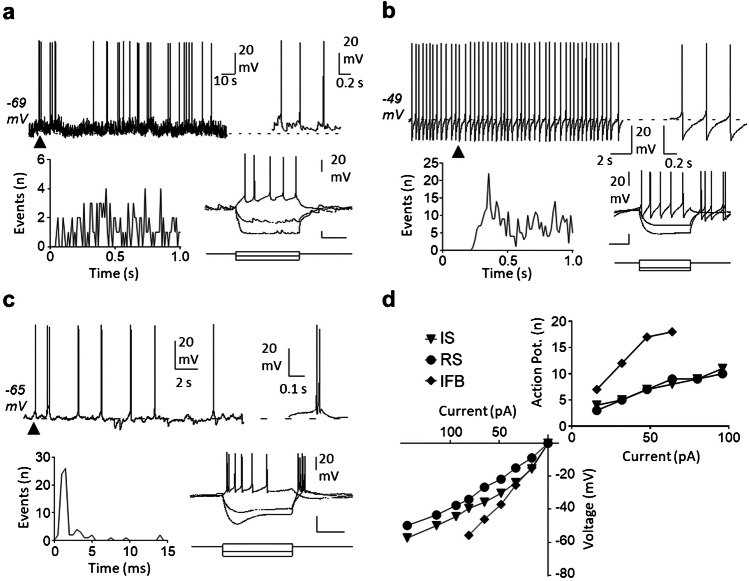
Intracellular records of neurons with spontaneous activity. Figure shows basic electrophysiological properties of three neurons with spontaneous activity recorded from spinal cord slices of adult mice with whole cell recording techniques. Original recordings are shown for neurons with irregular single spike (**a**), regular single spike (**b**), and irregular fast burst firing (**c**). Long unperturbed recordings are shown in the upper-left panels, followed by time expanded segments of the recordings marked by black triangles in the left. Resting membrane potential is indicated by dotted lines. Auto-correlograms are included below the original recordings to illustrate the firing pattern. Finally, responses to positive and negative current injection pulses are show for each neuron. Traces below the voltage recordings indicate the current intensity applied (calibration bars 100 pA; 200 ms). Graphs in (**d**) show a quantification of the voltage responses to negative current pulses measured at the maximum deflection for the three neurons (lower-left) and the action potential (Action Pot.) firing in response to positive current injections (upper-right)

Intracellular recordings from these different types of neurons are scarce at present [54, 79]. Figure 6 shows examples of voltage recordings from three different neurons. Regular single spike neurons tend to produce tonic firing to depolarizing current pulses and relatively scarce synaptic activity. Many irregular single spike neurons do show tonic firing as well but some of them show different degrees of firing accommodation to depolarization. In contrast, fast bursting neurons are highly excitable and show electrophysiological characteristics that favor their brisk behavior such as high resistance, initial bursts to depolarization, and rebound excitation at the end of hyperpolarizing current pulses. Although it is expected that electrophysiological traits support the observed spontaneous behavior, there is no strict correlation between them.

### Origin of spontaneous activity: External inputs or autogenous activity?

This is a recurrent question in the field. Steedman and Zachary studied responses of wide dynamic range neurons of anesthetized cats to light mechanical touch and to heat stimulation of their receptive fields and observed that touch tended to evoke burst-like responses, whereas heat triggered low frequency tonic discharges [87]. They also observed that spontaneous or background activity in these neurons “was stationary, and spikes occurred singly or occasionally in clusters.” After a detailed analysis, they concluded that this background activity was owing to random activity in large and small caliber afferent fibers. However, experiments from other laboratories demonstrated that acute sectioning of primary afferents does not eliminate spontaneous activity in all dorsal horn neurons [73]. Of course, nerve sectioning does not preclude the possibility that spontaneous activity arises from dorsal root ganglia or other remaining elements of sensory afferent fibers.

A number of investigations have studied the role of cortical areas and corresponding descending tracts on spinal cord activity [13, 18, 33, 73, 83, 85]. The combined output from these reports indicate that descending signals can modulate spontaneous activity by increasing or decreasing frequency or even changing activity patterns, but neither indicate that this input is necessary to sustain spontaneous activity.

Further evidence from recent investigations using in vitro preparations demonstrate that spontaneous activity can be recorded in isolated cords or even in slices of the cord [92]. Although this may be seen as a convincing demonstration that external input is not necessary, backfiring of primary afferents can be recorded in isolated preparations; therefore, it would be possible that central elements of the afferents retain activity after sectioning, or perhaps as a consequence of sectioning, and then they transmit this activity to the dorsal horn.

Perhaps the most compelling demonstration that spontaneous activity is not directly or exclusively sustained by any source of external input comes from pharmacological experiments using synaptic blockers for the main neurotransmitter systems known to operate in the cord. Several laboratories have shown that blocking GABA-, NMDA-, and peptidergic receptors produces an almost complete abolition of antidromic activity in dorsal roots [12, 53, 54, 102], suggesting that backfiring of afferents is owing to a mechanism presynaptic to the afferents.

Under synaptic blockade conditions, spontaneous activity is still recorded, but only in a subset of neurons [44, 58]. This indicates that there is an intrinsic source of excitability within the cord. Experiments in our laboratory indicate that neurons with spontaneous activity resistant to synaptic block are mostly those showing regular patterns [54]. In contrast, neurons with irregular patterns of spontaneous activity stop firing in the presence of synaptic blockers. Interestingly, we reported a few cases of neurons classed as irregular that become regular after synaptic blockade. We interpreted these observations as the impact of synaptic input on neurons with intrinsic membrane properties allowing rhythmic firing. This may cause confusion and may explain disagreement between experiments, particularly when comparing data obtained from in vivo and in vitro preparations, since the former condition is likely to exhibit greater synaptic activity.

At present, the general view on this issue is that spontaneous activity depends ultimately on the intrinsic membrane properties of a subgroup of neurons that allows for autogenous neuronal activity and that this intrinsic rhythmicity is modulated by synaptic input from different sources.

### Intrinsic ionic mechanisms generating autorhythmicity in dorsal horn neurons

Studies on ionic mechanisms responsible for spontaneous firing have focused traditionally on brain nuclei such as the basal ganglia, hippocampus, cerebellum, thalamus, and hypothalamus (see Harris-Warrick [35] for a review). The emerging view is that there is a wide array of mechanisms for autorhythmicity and that they are owing to the expression of a variety of ionic channels giving rise to currents that allow for generation, facilitation, and shaping of activity traits.

Generating currents are fundamental to allow crossing the threshold voltage and the actual firing of action potentials. Among them, the most commonly reported is the persistent sodium current or I_NaP_ [3, 8, 9, 11, 21, 22, 39, 71, 72, 96, 99]. However, the expression of I_NaP_ is not sufficient for spontaneous firing, since the current is expressed in silent neurons [42].

Additional currents may facilitate this process by maintaining resting potential close to the firing threshold. Hyperpolarization-activated current (I_H_), Ca^2+^-activated nonselective cation channels (ICAN), and leak sodium channels (NALCN) are likely involved in this role [10, 40, 48, 52, 55, 64, 67, 91].

Finally, other currents are necessary to maintain rhythmicity and to determine the number of action potentials per cycle. This seems to be the case for calcium-dependent potassium currents (I_KCa_) of the Small (SK) and Big (BK) conductance families, which alone or in conjunction with others (I_H_, L- and T-type calcium) [6] may shape different forms of rhythmicity. Voltage-dependent potassium currents appear to modulate firing frequency [22, 50, 65].

Ionic mechanisms of spontaneous firing in spinal cord neurons have received little attention compared with neurons in other areas of the CNS. However, the principal ionic currents under discussion have been reported in neurons of the spinal cord.

Persistent sodium currents are present in dorsal horn neurons from neonatal and adult rodents, where they can influence repetitive firing properties and the integration of synaptic inputs [44, 54, 70, 82]. NALCN currents have also been reported in spinal cord neurons and may regulate intrinsic excitability and responsiveness to substance P in projection neurons [28].

Voltage-activated calcium channels of both low and high voltage activated families are expressed by dorsal horn neurons [81]. L-type calcium channels play a fundamental role in shaping the firing properties of some dorsal horn neurons by supporting plateau potentials in deep and superficial dorsal horn neurons [26, 66].

Several voltage dependent potassium channels are also expressed in dorsal horn neurons, where they have a profound impact on excitability under physiological and pathological conditions [36, 61, 74, 75].

Finally, the hyperpolarization activated current, I_H_, is expressed in a considerable proportion of dorsal horn neurons in both adult and neonatal animals [54, 77]. In addition, the I_H_ is coexpressed with T-type calcium currents in some neurons, where they shape the rebound firing and spike timing precision in these neurons [76].

Baccei’s group has made a consistent analysis of ion currents expressed in dorsal horn neurons with regular bursting patterns. This pattern was found in interneurons and projecting neurons of the dorsal horn of newborn animals. Blocking I_NaP_ with riluzole eliminated spontaneous activity in some of them, but not all [44, 45]. They reported that neurons with spontaneous firing have a higher I_NaP_ to leak ratio than silent neurons and may possess lower K_ir_ currents [44, 46], so that blocking K_ir_2 could turn silent neurons into bursting mode. Blocking calcium channels of the N- and L-type, but not T-type, depolarized membrane potential and reduced the frequency of bursts [44]. In addition, the occurrence of bursts was severely impaired after blocking I_KCa_ or intracellular dialysis with BAPTA. Additionally, blocking ICAN with flufenamic acid reduced the plateau potentials regulating burst duration [44]. All these observations suggest that many of the currents that are relevant for shaping spontaneous activity in different areas of the brain have similar roles in the spinal cord and that many different mechanisms for fine-tuning of this activity are possible.

We have shown that synaptic blockade eliminates spontaneous activity in dorsal horn neurons with irregular firing patterns, whereas activity persists in a majority of neurons with regular patterns [54]. A large percentage of these neurons resistant to synaptic blockade (≈75%) ceased firing in the presence of ZD7288, a blocker of the I_H_ current. Similarly, a considerable percentage of resistant neurons ceased firing in the presence of riluzole at low concentrations. Riluzole is not specific for I_NaP_ and may affect some neurotransmitter systems; however, once neurotransmission has been blocked with specific tools, the effects of riluzole can be attributed to I_NaP_ blockade. When both compounds where perfused together, 90% of neurons with spontaneous activity resistant to synaptic block ceased firing. These observations suggest an important role for persistent sodium and I_H_ currents at sustaining intrinsic firing in spinal cord neurons. Interestingly, combined perfusion of riluzole and ZD7288 on preparations without synaptic block produced a dramatic reduction of spontaneous activity in neurons of all classes, including irregular firing patterns. This observation supports a central role for autorhythmic neurons in sustaining spontaneous activity in the dorsal horn.

### Toward a model circuit sustaining spontaneous activity in the dorsal horn

**Fig. 7:**
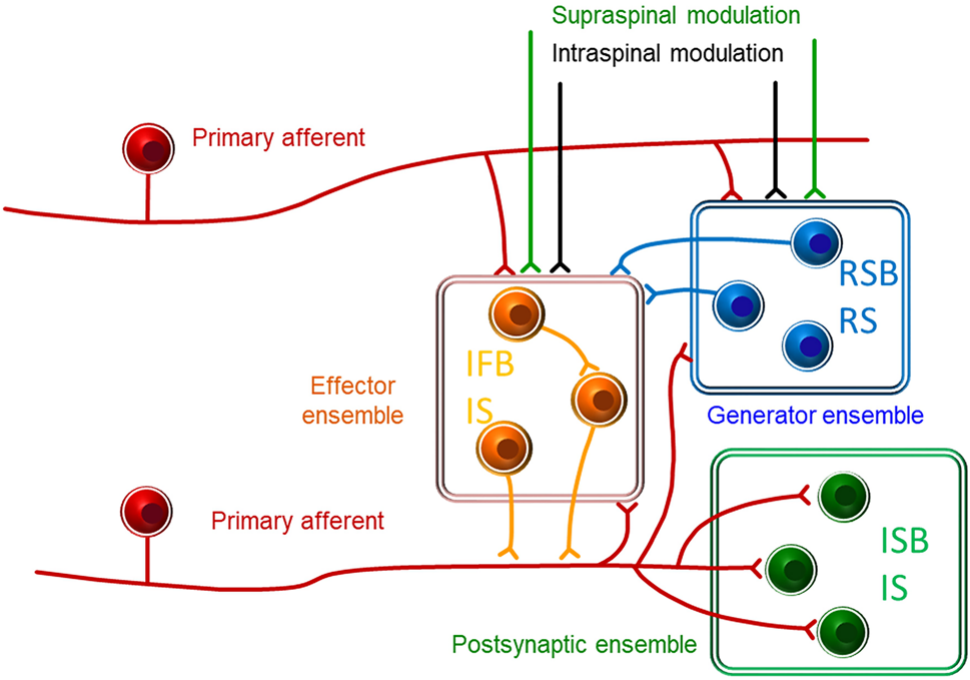
Proposed arrangement of neurons in the dorsal horn to explain the origin and spread of spontaneous activity in the cord. A generator ensemble of neurons (blue) provides synaptic input to an effector ensemble (orange) that excites primary afferents. Both generator and effector ensembles are influenced by descending and intraspinal modulation. Finally, primary afferents spread excitation to an ensemble of postsynaptic neurons formed by projection neurons and interneurons. *IS* Irregular single spike, *IFB* irregular fast burst, *ISB* irregular slow burst, *RS* regular single spike, *RSB* regular slow burst

On the basis of the previous discussion, we proposed a basic model circuit to explain the elements and connectivity involved in the generation of spontaneous activity in the dorsal horn [53]. This circuit requires a source of synaptic drive provided by neurons with spontaneous rhythmic activity located in the dorsal horn. This first ensemble of neurons would work as an internal generator of the spinal cord and probably several such generators may be found in a single cord segment [92]. Another element of the circuit would be involved in spreading excitation. This element of the system may involve irregular neurons, some of which excite primary afferents. Recent experiments in our laboratory suggest that these are mainly irregular single spike and fast burst neurons. Action potentials transmitted antidromically in primary afferents would explain the activity recorded in dorsal roots after transection. Action potentials transmitted orthodromically through afferent terminals would spread activity to many other dorsal horn neurons working as an amplifier [12]. Under physiological conditions, spontaneous activity would be modulated by afferent and descending inputs. Figure 7 shows these ideas in a cartoon modified from Lucas-Romero et al. [53].

### Function of spontaneous activity in the maturation of dorsal horn circuits

Spontaneous activity has been proposed as a key element in the development of CNS in a variety of animal species and many issues related to this view have received experimental support (see an extended review on the topic by Baines and Landgraf) [5].

Manipulation of spontaneous activity in particular critical periods of the embryonic development may delay the development toward mature systems or even produce permanent changes in their function. Sensory feedback resulting from spontaneous movements is instrumental for coordination of activity in developing sensorimotor spinal cord circuits in neonatal rats [38]. Similarly, recent studies in ventral nerve cords of drosophila (equivalent to the spinal cord of mammals) show that spontaneous activity in neonatal neurons tend to produce muscle activity and the resulting mechanical sensory feedback helps with the fine tuning of locomotor networks [16].

Development of sensory systems also benefit from spontaneous activity prior to actual sensory experience. This has been demonstrated for the different sensory channels and across species [43]. Whereas in early developing systems spontaneous activity comes in highly synchronic waves in which many neurons intervene mainly connected through gap junctions, mature systems tend to show less synchronous activity. Although the mechanisms underlying this phenomena and its function is not completely clear, a consensus view is that spontaneous activity contributes toward a correct wiring of sensory systems through a Hebbian process.

A second step in the maturation of sensory systems is early sensory experience, which may contribute toward a fine-tuning of sensory circuits. Li and Baccei found a significant number of neurons with membrane properties favoring intrinsically generated firing of action potentials in the dorsal horn of neonatal rats [44]. These neurons were present in lamina I, mainly concerned with nociceptive processing, and their numbers appeared to decrease toward the second week of life. The authors suggested that the spontaneous activity generated by these neurons may assist in the fine tuning of spinal local circuits controlling the nociceptive withdrawal reflexes in the absence of actual nociceptive input.

Although this proposal is very interesting and well supported by a wide background of evidence that applies to all sensory systems and the CNS in general, it may be only part of the whole story since many groups (as reviewed above) have described neurons with intrinsic firing properties in the superficial and deep dorsal horn of adult animals. Therefore, other additional or complementary functions should be explored.

### Function of spontaneous activity in the maintenance of excitability

An additional role for spontaneous activity could be maintaining an appropriate level of excitability in spinal circuits. The level of excitability in the dorsal horn has to be maintained within a physiological range in order to generate responses (motor or sensory) adapted to the intensity and nature of external stimuli as well as the internal state of the system. Hyperexcitability of spinal neurons and circuits leads to increased pain perception and reflex activity, whereas hypoexcitability leads to anesthetic-like states.

A correct level of excitability is the result of a complex interplay between a variety of mechanisms. These include at least the following:

1. Ion conductances are essential to produce autorhythmicity in a subset of dorsal horn neurons. In addition, different ion conductances regulate membrane excitability of individual neurons modulating responses to their synaptic input.
2. Synaptic strength between particular excitatory or inhibitory synapses will determine, together with intrinsic properties, the responses of dorsal horn circuits to afferent input.
3. Interactions between neurons and glial cells may influence any of the previous.
4. Afferent input from the skin, muscles, joints, and viscera are the main source of external input to the spinal cord. Alterations in afferent input may induce homeostatic changes in excitability via modulation of ionic currents and synaptic strength [75].
5. Descending input from supraspinal centers constitute an additional and variable source of external input that adapts a correct level of excitability to varying external and internal conditions.

In the absence of external inputs, the local circuits are capable of sustaining spontaneous activity as previously reviewed. Therefore, we can speculate that the spontaneous activity generated by dorsal horn circuits constitutes the core mechanism of excitability upon which may impinge either brief afferent signals ultimately caused by peripheral stimulation or modulatory descending tonic signals that allow adaptation of excitability to different conditions such as sleep or physical stress.

### Is spontaneous activity of dorsal horn neurons a mechanism underlying sensory processing?

In the recent literature, there is a strong tendency to interpret spontaneous activity as a decisive element in the processing of sensory information and cognitive processes in general [4]. The metabolic consumption of the nervous system is high with little difference between rest and task engagement. Since most of the energy goes into the maintenance of spontaneous activity at rest, many authors believe that this activity has to be necessarily important for information processing; otherwise, evolution would have selected alternative and less expensive processing models [69]. Most of the studies investigating the processing function of spontaneous activity refer to the brain, particularly to the neocortex, which has an organization and function quite different from the spinal cord.

There is good agreement about the fact that perceptual processes involve at least two different types of mechanisms, i.e., bottom-up and top-down mechanisms. The first of them is related to the actual recollection of sensory information which occurs at sensory receptors and peripheral elements of sensory systems. The second is a complex set of elements formed by memories of past perceptions, expectations due to contextual clues, and the present state of the brain. Spontaneous activity in brain structures is generally associated with one or several of these top-down elements, although there is no comprehensive model explaining the specific function of this phenomenon at present.

Although there is no systematic analysis of the function of rhythmicity in neurons of the dorsal horn, several groups have provided hints that have been discussed above. Sandkühler and Eblen-Zajjur proposed that the dominant firing frequency of rhythmic neurons may not be a main signaling clue but changes in firing pattern “might act as novelty detectors or as gates opening and closing sensory channels” [84]. This is consistent with the hypothesis of inverted neurons from Cervero et al. [17]. We have also mentioned how different patterns of spontaneous activity in spinothalamic neurons may resonate with specific afferent inputs [98].

Additional and new ideas may come from studies in different brain areas since the nervous system often uses similar strategies to solve processing problems of different nature. However, in the absence of experimental evidence, ideas remain speculative.

### Spontaneous activity of dorsal horn neurons during pain states

Pain signals are processed in the spinal cord by two well differentiated types of neurons. Projecting nociceptive specific neurons are mainly located in superficial lamina I of the dorsal horn and receive inputs from nociceptive primary afferents. This is a “labeled line” for pain processing. Projecting wide dynamic range neurons have their somas in deeper laminae and receive convergent input from different afferent types carrying nociceptive and tactile signals. They code well for stimulus intensity but not for sensory quality. Some authors suggest that wide dynamic range neurons convey information on the magnitude of the nociceptive stimuli, whereas nociceptive specific neurons are involved in homeostatic and affective responses to the nociceptive stimuli [62].

A recent review analyzes the effect of different pain-inducing treatments (inflammation, arthritis, and neuropathy) on induced and spontaneous activity of wide dynamic range neurons of the dorsal horn [100]. The authors found that the coding capacity of stimulus intensity in these neurons is altered by inflammation but not by neuropathy. Instead, authors systematically report increased ongoing firing frequency in wide dynamic range neurons for both conditions, suggesting that this activity may contribute to sustaining spontaneous pain. Interestingly, nociceptive specific neurons do not seem to increase spontaneous activity under inflammatory conditions but do show increased responses to nociceptive stimuli [41]. These observations also apply to identified projecting spinothalamic neurons [23] and not just to interneurons.

Recently, we obtained experimental evidence supporting the idea that spontaneous activity undergoes subtle changes following peripheral inflammation [53]. Superficial dorsal horn neurons recorded in vitro from animals that had been pre-treated with an inflammatory procedure prior to cord extraction do not show substantial changes in spontaneous firing frequency compared to controls. However, cords from these pre-treated animals showed enhanced population bursts because neurons tend to fire their action potentials more often in synchronous events. This may increase the probability of antidromic firing in primary afferents and the ongoing firing frequency of spinal neurons downstream in the transmission chain. It is important to note that experimental inflammation increases backfiring in somatosensory afferents following inflammation [49, 92] and that this mechanism may contribute to explaining the origin of neurogenic inflammation, secondary hyperalgesia, and allodynia [19, 97].

## Concluding remarks

The literature reviewed shows that spontaneous activity is found in spinal cords of live animals of different species as well as in vitro preparations and that it comes in a variety of different patterns. We proposed a comprehensive classification that may serve for reference in future studies, which includes regular and irregular patterns as well as single spiking vs bursting behavior, both variables consistently found in experimental studies. This classification is wide enough to cover the variety of patterns reported in the literature and it breaks down bursting behavior in several different categories.

We also reviewed the origin of spontaneous activity and proposed a basic circuit to maintain excitability in the dorsal horn allocating a role for regularly firing neurons as generators and irregularly firing neurons as transmitters of activity.

We find an important lack of experimental evidence regarding function of spontaneous activity in physiological and pathological states. Although a role during development is well justified, other possible roles during adulthood are not understood yet and further investigation of this issue is required. Particularly, its possible role in sensory information processing remains speculative.

## Data Availability

No datasets were generated or analysed during the current study.
